# A Stability-Indicating HPLC Method for the Determination of Memantine Hydrochloride in Dosage Forms through Derivatization with 1-Fluoro-2,4-dinitrobenzene

**DOI:** 10.3797/scipharm.1310-09

**Published:** 2013-12-09

**Authors:** Hassan Jalalizadeh, Mahdi Raei, Razieh Fallah Tafti, Hassan Farsam, Abbas Kebriaeezadeh, Effat Souri

**Affiliations:** 1 Department of Research & Development, Osvah Pharmaceutical Co. Tehran, Iran.; 2 Department of Medicinal Chemistry, Faculty of Pharmacy, Tehran University of Medical Sciences, Tehran, Iran.; 3 Department of Toxicology and Pharmacology, Faculty of Pharmacy, Tehran University of Medical Sciences, Tehran, Iran.; 4 Department of Pharmacoeconomy and Pharmaceutical Administration, Faculty of Pharmacy, Tehran University of Medical Sciences, Tehran, Iran.

**Keywords:** Memantine, Dinitrofluorobenzene, HPLC, UV-detection, Dosage forms

## Abstract

Memantine is chemically a tricyclic amine and is used for Parkinson’s disease and movement disorders. Although several HPLC methods with different derivatization reagents have been developed for the determination of memantine in biological fluids, there are some complications which limit the use of these methods in routine analysis of memantine in *in vitro* tests. We established a simple, sensitive, precise, and accurate HPLC method for the quantification of memantine in dosage forms. Pre-column derivatization of memantine was performed with 1-fluoro-2,4-dinitrobenzene and the reaction product was separated on a Nova-Pak C18 column. A mixture of acetonitrile and sodium dihydrogenphosphate (pH 2.5; 0.05 M) (70: 30, v/v) was used as the mobile phase. UV detection was performed at 360 nm. Forced degradation studies were performed on a powdered tablet sample of memantine hydro-chloride using acidic (0.1 M hydrochloric acid), basic (0.1 M sodium hydroxide), oxidative (10% hydrogen peroxide), thermal (105°C), photolytic, and humidity conditions. Good linearity (r^2^=0.999) was obtained over the range of 1–12 μg mL^−1^ of memantine hydrochloride with acceptable within-day and between-day precision values in the range of 0.05–0.95%. The proposed method was used for the assay determination and dissolution rate study of memantine dosage forms with excellent specificity.

## Introduction

**Fig. 1:**
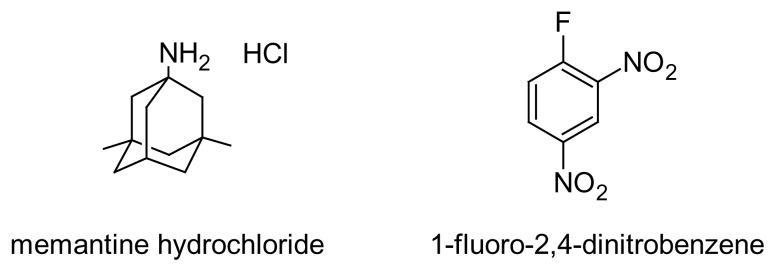
Chemical structure of memantine and 1-fluoro-2, 4-dinitrobenzene

Memantine hydrochloride, 1-amino-3,5-dimethyladamantane hydrochloride (Figure 1), is chemically a tricyclic amine and is pharmacologically related to the antiviral prototype amantadine and its α-methyl derivative, rimantadine. Memantine and rimantadine have been approved in the U.S. for the prophylaxis and treatment of influenza. Amantadine and memantine are also used for the treatment of Parkinson’s disease and movement disorders [1]. Memantine is also approved for the treatment of moderate-to-severe Alzheimer’s disease [2], and has now received a limited recommendation by the UK’s National Institute for Clinical Excellence for patients who fail with other medicinal therapeutic options [3].

Memantine has no significant ultraviolet, visible, or fluorescence absorption. Studies requiring the quantitation of memantine in plasma, cerebrospinal fluid, urine, and other tissues have used gas chromatographic-mass spectrometric (GC-MS) [4], liquid chromatography-tandem mass spectrometry [5–9], and HPLC methods in combination with ultraviolet detection or fluorescence detection using different derivatizing reagents [10–17]. The reported methods could be used for the determination of memantine in dosage forms, but there are some disadvantages. The fluorescent OPA-drug derivative is relatively unstable and should be injected immediately after the preparation of samples [10]. The derivatization of memantine with dansyl chloride is simple, but this derivatization method is a time-consuming process [11]. The reaction time for the derivatization of memantine and 4-fluoro-7-nitro-2,1,3-benzoxadiazole is not too long (about 5 min), but the total run time of the HPLC method is about 40 min [12].

Recently, a validated stability-indicating HPLC assay for the determination of memantine hydrochloride with UV-detection using pre-column derivatization by 9-fluorenylmethyl chloroformate (FMOC) has been developed by Narola et al. [18]. The linearity of this method was in the range of 70–130 μg mL^−1^ which is not suitable for the quantification of low concentrations of memantine hydrochloride, especially in dissolution media for the evaluation of drug formulations. The purpose of the present study was to develop a sensitive, accurate, and precise HPLC method for the quantitative determination of memantine following the derivatization with 1-fluoro-2,4-dinitrobenzene (FDNB) (Figure 1). The FDNB reagent has been used before for the determination of some other drugs [19–22]. The proposed method could be used for the determination of memantine in pharmaceutical dosage forms, especially in dissolution media with UV-detection for drug quality control purposes.

## Results and Discussion

### Derivatization Reaction

In the present method, pre-column derivatization with FDNB was used for the analysis of memantine. The reaction of FDNB with the primary amines was performed in a mild alkaline medium using a borate buffer (pH=9.1). In this condition, FDNB had hydrolyzed to dinitrophenolate, which could be converted to dinitrophenol under acidic conditions at the end of the derivatization procedure [23]. The maximum absorbance of the FDNB derivative at 360 nm was used as the HPLC analytical wavelength.

**Fig. 2:**
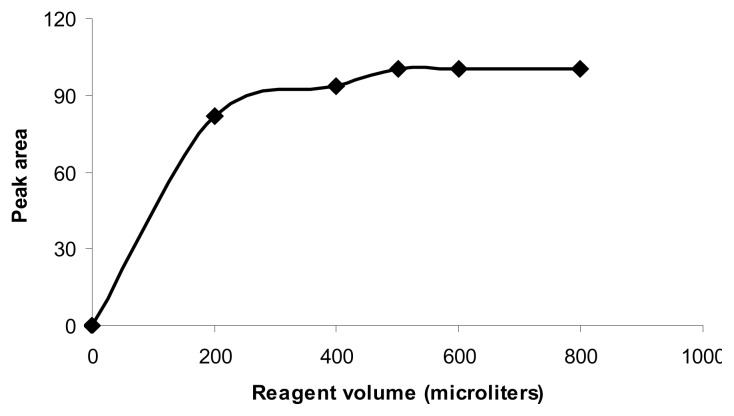
Effect of reagent volume (6 μg mL^−1^) (30°C for 30 min) on the derivatization reaction of FDNB and memantine (n=3)

It was shown that the reaction was completed by using 500 μL of the reagent (6 μg mL^−1^) (Figure 2). Higher amounts of the reagent did not increase the reaction yield, but showed interfering byproducts.

**Fig. 3:**
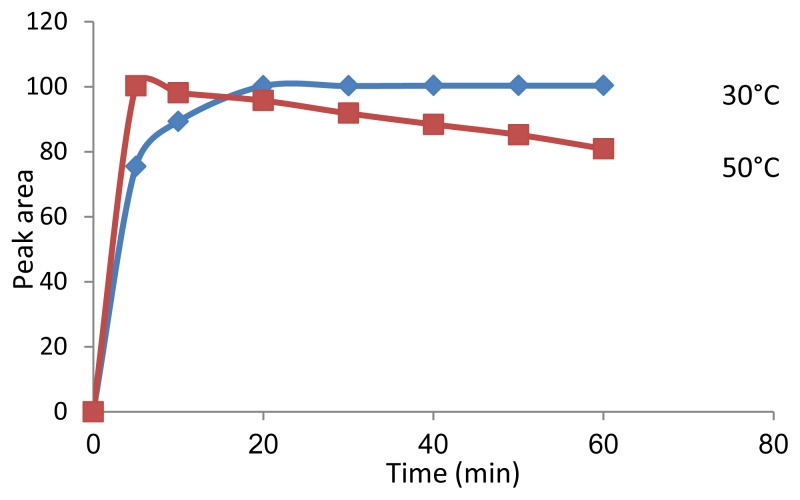
Effect of time and temperature on the derivatization reaction of FDNB and memantine using 500 μL of FDNB reagent (6 μg mL^−1^) (n=3)

By performing the derivatization reaction in different temperatures, it was shown that the reaction was completed after 30 min at 30°C or 5 min at 50°C (Figure 3). The reaction product showed more stability in lower temperatures. On the other hand, no reaction was achieved at room temperature. The best results were obtained at 30°C within 30 min.

### Chromatographic Conditions

**Fig. 4:**
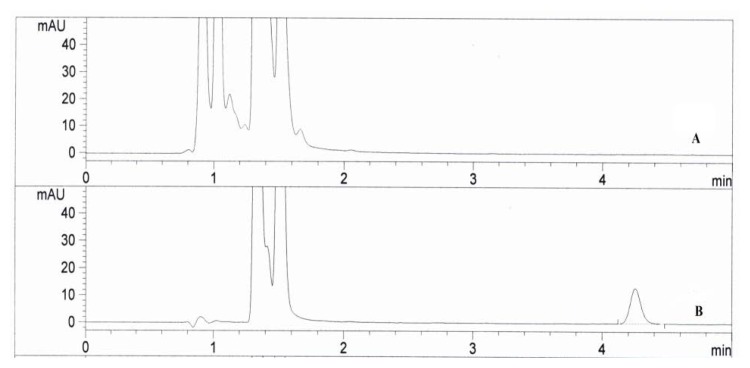
Typical HPLC chromatograms of: (A) Blank Solution; (B) Standard solution of memantine hydrochloride and FDNB product in optimized conditions (500 μL FDNB, 6 μg mL^−1^, at 30°C for 30 min).

To find out the best chromatographic conditions, a Nova-Pak C18 column and varying mobile phase systems were used. Good baseline resolution, minimal tailing, and peak sharpness were observed by using a mixture of acetonitrile and sodium dihydrogen-phosphate (pH 2.5; 0.05 M) (70: 30, v/v) as the mobile phase. Suitable chromatographic specificity with no interference from the reagent byproducts, dosage form excipients, or dissolution medium at the retention time of memantine derivative was observed. An acceptable retention time and peak reproducibility for the memantine derivative was achieved. The memantine derivative was well-resolved and eluted at a retention time of about 4 min and the total run time was within 6 min. The short run time of the proposed HPLC method is suitable for multi-sample analysis programs. Representative chromato-grams are shown in Figure 4.

### System Suitability Test

The suitability of the HPLC method was verified by repeated injections and the theoretical plates, symmetry, and repeatability of the retention time and peak area were determined. The results and the acceptable limits are shown in Table 1.

### Forced Degradation Studies

All of the stressed samples were subjected to the derivatization reaction and injected into the HPLC system according to the described chromatographic conditions. The results of forced degradation in different conditions are presented in Table 2.

**Fig. 5:**
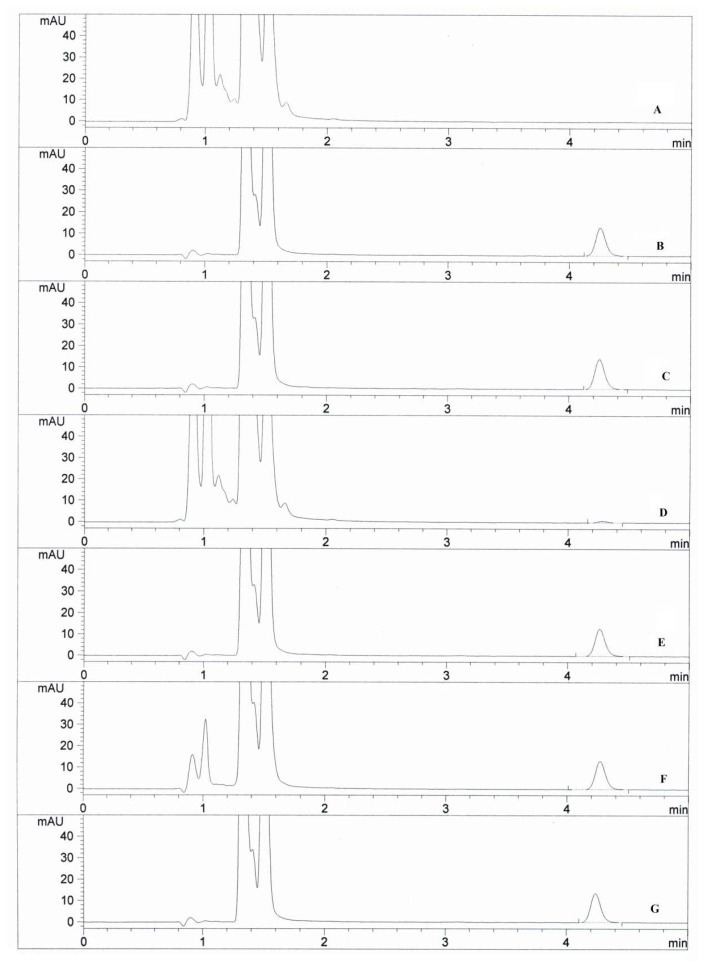
Typical HPLC chromatograms of the reaction product of memantine and FDNB under optimized conditions (500 μL FDNB, 6 μg mL^−1^, at 30°C for 30 min): (A) Blank solution; (B) Powdered tablet in 0.1 M HCl after 1 h at 70°C; (C) Powdered tablet in 0.1 M NaOH after 1 h at 70°C; (D) Powdered tablet in 10% H_2_O_2_ after 1 h at 70°C; (E) Powdered tablet after 5 days exposure to 105°C dry heat; (F) Powdered tablet after 5 days exposure to photolytic chamber; (G) Powdered tablet after 5 days exposure to humidity.

Degradation was found to be relatively slow in acidic, basic, heat, light, and humidity stress conditions. On the other hand, complete degradation in oxidative conditions was observed. No degradant peak was detected in the forced degradation study chromatograms. The typical chromatograms of drugs under stress conditions are shown in Figure 5.

### Linearity

Six series of standard memantine hydrochloride solutions in the range of 1–12 μg mL^−1^ were determined under the optimized conditions and the calibration curves were constructed. The calibration curves were found to be linear as shown by the equation presented in Table 3. Statistical data are also presented in Table 3.

### Accuracy and Precision

The synthetic samples of memantine hydrochloride at 1, 6, and 12 μg mL^−1^ on one day and three separate days were used to find out the accuracy and precision of the method. Within-day and between-day data are given in Table 4. The within-day and between-day R.S.D. values were less than 1.2% in all three selected concentrations.

To find out the ruggedness of the method, the within-day and between-day data for the determination of memantine hydrochloride samples by two analysts using two different HPLC systems were compared. No significant variation was observed (R.S.D. < 2%).

The robustness of the developed method was checked by studying the effect of minute changes in the mobile phase composition (±10%) and buffer pH (±0.3) on the chromatographic factors. The peak area of the reaction product was not influenced (less than ±0.98%) by changing the composition of the mobile phase or pH of the phosphate buffer. According to the results shown in Table 5, the retention time of memantine was insignificantly influenced (1.1%) by changing the pH of the phosphate buffer, whereas it was considerably affected (about 17.1%) by changing the mobile phase composition.

### Specificity

**Fig. 6:**
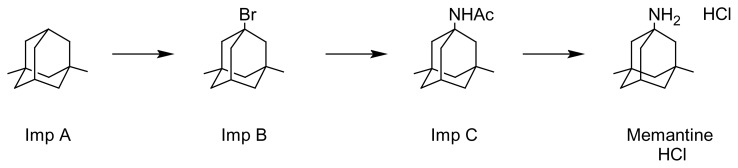
Synthetic scheme of memantine hydrochloride

It was shown that the inactive ingredients from the tablets did not interfere in the drug peak. Furthermore, process-related impurities of memantine, namely, Imp-A, Imp-B, and Imp-C (Figure 6) could not react with the derivatization reagent due to the lack of an amino functional group. Hence there would not be any peak interference between memantine and its process-related impurities in the drug substances using the developed HPLC method. Thus the proposed HPLC method would be useful to quantify memantine hydrochloride in dosage forms in the presence of excipients and related substances.

### Sensitivity

The limit of quantification with a R.S.D. <1.2% was found to be 1 μg mL^−1^ for memantine hydrochloride. The limit of detection with a S/N ratio of 3 was found to be 0.2 μg mL^−1^.

### Solution Stability

Comparison of stock solutions of memantine hydrochloride after 7 days at 4°C with freshly prepared samples showed no significant changes (±1%). The memantine-DNFB derivative was also found to be stable at least for 72 h.

### Assay and Dissolution Test of Ebixa® 10 mg Tablets

**Fig. 7:**
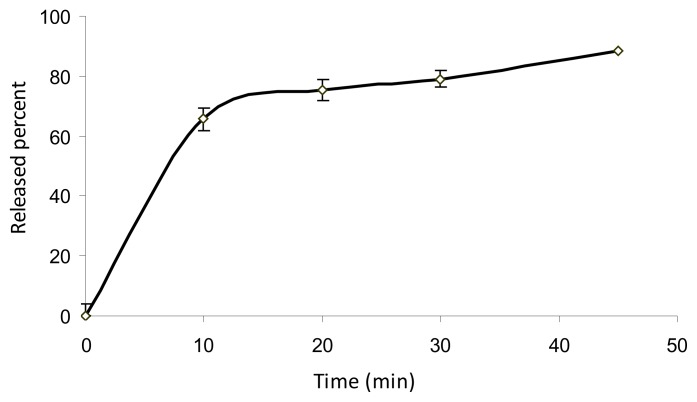
Dissolution profile of Ebixa® 10 mg tablets (n=6) using 0.1 M HCl and 2 g L^−1^ NaCl at pH=1.2 as dissolution medium and basket at 100 rpm after derivatization under optimized conditions (500 μL FDNB, 6 μg mL^−1^, at 30°C for 30 min)

The assay results using the proposed method for the determination of Ebixa® tablets showed good agreement with the labeled amount (10.13±0.19 mg). The validated method was also successfully used to study the dissolution profile of Ebixa® tablets. Based on the results in Figure 7, more than 85% release of the drug was observed within 45 min.

## Conclusion

The present developed HPLC method is a simple, accurate, sensitive, and reproducible as well as economical method for the analysis of memantine hydrochloride in dosage forms, which could be used for routine *in vitro* tests. In this report, FDNB has been used as a derivatizing reagent for the first time for the determination of memantine. The most outstanding advantages of this method include the usage of an inexpensive derivatizing reagent, suitable derivatization time, and stability of the reaction product. Also, there is no need for prior separation or purification before analysis as the byproduct of the reagent shows different polarity with the reaction product and could be easily separated from the reaction product. Also, a common HPLC system (isocratic system, UV detector, and ambient temperature) is used for the separation of the reaction product. The total run time of this method is relatively short, which is appropriate for the determination of multiple samples in a short period of time and the proposed method would be a useful and suitable method for quality control studies.

## Experimental

### Chemicals

Memantine hydrochloride was obtained from Industrial Chemica, India, (Batch No: PR110650). Ebixa® 10 mg tablets, Lundbeck, Denmark (Batch No: 0022035) were prepared from a local market. 1-fluoro-2,4-dinitrobenzene (FDNB) (purity >99%), the derivatization reagent, was purchased from Fluka (Switzerland). Acetonitrile was HPLC grade and purchased from Merck (Darmstadt, Germany). All other analytical grade chemicals were purchased from Merck. Ultra pure water was prepared using a Milli-Q purification system (Millipore, Milford, MA, USA).

### Instrumentation

A Shimadzu double beam UV–visible spectrophotometer (UV-160, Shimadzu, Kyoto, Japan) was used for the spectrophotometric measurements. The HPLC system consisted of a Quaternary Pump, Degasser G1322A, and a variable UV Detector G1314B all from 1200 series Agilent Technology (Hewlett-Packard-Strasse 876337 Waldbronn, Germany). The data acquisition system was multi-channel Agilent Chemstation software for chromatography, version B.02.01 [244]. A Metrohm pH meter (Model 691, Switzerland) was used for pH adjustments.

### Standard Solutions

An accurate amount of memantine hydrochloride was dissolved in 0.1 M HCl to reach a final concentration of 100 μg mL^−1^. Standard working solutions of memantine hydrochloride at 1, 2, 4, 6, 8, 10, and 12 μg mL^−1^ were prepared by subsequent dilutions. By dissolving 600 μL of the FDNB reagent in 100 mL of acetonitrile, a solution of 6 μg mL^−1^ (0.048 M) was prepared. Notice that this reagent should be handled carefully since it is a skin irritant. All these solutions were stored at 4°C until use. A borate buffer (0.25 M) was prepared by dissolving appropriate amounts of H_3_BO_3_ and KCl in water and adjusting the pH value to 9.1.

### Sample Preparation

The derivatization process was carried out by transferring 1 mL of each standard solution of memantine hydrochloride into 10 mL measuring flasks. Then, 2 mL of the borate buffer, 500 μL of the FDNB reagent (6 μg mL^−1^), and 3.6 mL of acetonitrile were added to each flask. The mixtures were vortexed for 10 s and kept at 30°C for 30 min. To stop the derivatization process, 300 μL of 1 M HCl solution were added after cooling to room temperature and the volumetric flasks were made up to volume with acetonitrile. Fifty microliters of the resulting solution was injected into the HPLC system.

### Chromatographic Conditions

The analytical separation of memantine was achieved using a Nova-Pak® C18, 4 μm column (250 mm×4.6 mm, Waters, Milford, MA, U.S.A.). The mobile phase consisted of a mixture of acetonitrile and sodium dihydrogenphosphate (pH 2.5; 0.05 M) (70: 30, v/v). The mobile phase was passed through a 0.45 μm Millipore membrane filter and ultrasonicated for 10 min for degassing. The flow rate was set at 1.5 mL min^−1^ with UV detection at 360 nm. All analytical separations were conducted at room temperature.

### Optimization of Reaction Conditions

A standard solution of memantine hydrochloride at 6 μg mL^−1^ was used for the optimization of the derivatization reaction. The effect of the reagent volume in the range of 200–800 μL and also temperature (30 and 50°C) were studied on the derivatization reaction. Optimized derivatization conditions were ultimately obtained using 500 μL of the reagent solution (6 μg mL^−1^) and 30 min at 30°C.

### Forced Degradation Studies

Forced degradation of memantine hydrochloride in different conditions was performed using powdered tablet samples.

#### Acid, Base, and Oxidative Degradation

Memantine hydrochloride solutions were prepared in 10% H_2_O_2_, 0.1 M HCl, and 0.1 M NaOH. The resulting mixtures were heated for 1 h in a water bath at 70ºC in order to accelerate the degradation of the compound. A sample solution was prepared at 10 μg mL^−1^ concentration value of memantine hydrochloride and injected to the HPLC system after derivatization.

#### Thermal Degradation

In order to perform the temperature stress studies, 20 tablets were exposed to dry heat (105°C) in an oven for 5 days. The tablets were removed from the oven and weighed, crushed, and mixed in a mortar and pestle. An aliquot of powder equivalent to one tablet was then prepared for analysis using the proposed analytical method at the 10 μg mL^−1^ final concentration of memantine hydrochloride.

#### Photolytic Degradation

A photolytic degradation study was carried out on tablet powder by exposing the sample to visible light in a photolytic chamber at 2600 Lux for 5 days, followed by the analysis of the sample according to the proposed analytical method after appropriate dilution to 10 μg mL^−1^ final concentration of memantine hydrochloride.

#### Humidity Degradation

An amount of tablet powder was subjected to humidity degradation by keeping it at 25°C and 90% RH for 5 days, followed by the analysis of sample according to the proposed analytical method at the 10 μg mL^−1^ final concentration of memantine hydrochloride.

The peak areas of memantine in all different degradation conditions were compared with freshly prepared samples at the 10 μg mL^−1^ concentration by the proposed analytical method and the percentage of degradation were calculated.

### Calibration Curve

Standard calibration solutions were prepared with six calibrators over the memantine hydrochloride concentration range of 1–12 μg mL^−1^ by subsequent dilution of the standard solution. The derivatization process and HPLC analysis were performed as described above. Construction of the calibration curves were achieved by plotting the measured peak areas of derivatized memantine versus the concentrations of standard samples and the statistical data was calculated.

### Precision and Accuracy

To establish the within-day and between-day precision and accuracy, three different solutions of memantine hydrochloride at three different concentration values (1, 6, and 12 μg mL^−1^) were prepared in triplicate and analyzed according to the general procedure. This procedure was repeated for three separate days.

### Application of the Method

For the determination of memantine hydrochloride in tablet dosage forms, 20 Ebixa® tablets were weighed and finely powdered. An appropriate portion of powder equivalent to about 10 mg of memantine hydrochloride was accurately weighed and transferred to a 100 mL volumetric flask. The flask was made up to volume with 0.1 M HCl and sonicated for 15 min. Then, the solution was passed through a 0.45 μm Millipore membrane filter and diluted with 0.1 M HCl to reach a final concentration of about 10 μg mL^−1^. After the derivatization process, the drug concentrations of six replicate assay solutions were determined by HPLC using the constructed calibration curve.

### Dissolution Studies

Drug dissolution studies of Ebixa® tablets were evaluated by using a dissolution apparatus (Erweka DT 820, Heusenstamm, Germany). The apparatus consisted of eight vessels in a warm bath at 37±0.5°C. 900 mL of 0.1 M HCl containing 2 g L^−1^ NaCl at pH 1.2 was used as the dissolution medium. The basket apparatus at 100 rpm was used for measuring the dissolution rate of the tablets. Samples of 4 ml were withdrawn at predetermined time intervals at 10, 20, 30, and 45 min. Sample volumes were replaced by pre-warmed dissolution media at 37°C in order to maintain the sink conditions. The solutions were subjected to the derivatization method after passing through a 0.45 μm Millipore membrane filter according to the abovementioned method.

## Figures and Tables

**Tab. 1:** System suitability parameters

Parameters	Found	Acceptable limits
Theoretical plates (n=3)	1853	N> 1500
Asymmetry (n=6)	1.28	T< 1.5
Repeatability (t_R_) (n=6)	0.255	R.S.D.< 1%
Repeatability (Peak area) (n=6)	0.473	R.S.D.< 1%

t_R_: Retention time (min); N: Theoretical plate; T: Tailing factor; R.S.D.: Relative Standard Deviation

**Tab. 2:** Results of forced degradation

Stress condition	% Degradation	S.D.	%R.S.D.
Control	0	–	–
Digested with 0.1M HCl solution for 1h at 70ºC	20.68	0.05	0.22
Digested with 0.1M NaOH solution for 1h at 70ºC	14.97	0.06	0.38
Digested with 10% H_2_O_2_ solution for 1h at 70ºC	95.31	0.44	0.46
Dry heated for about 5 days at 105ºC	21.27	0.03	0.15
Exposed to visible light about 2600 Lux for 5 days	19.21	0.06	0.29
Exposed to relative humidity 95% at 25 ºC for 5 days	17.44	0.07	0.40

**Tab. 3:** Statistical data of calibration curves of memantine hydrochloride in standard solutions

Parameters	Memantine hydrochloride
Linearity	1–12 μg mL^−1^
Regression equation	Y = 11.737× − 0.973
Standard deviation of slope	0.0379
Relative standard deviation of slope (%)	0.3233
Standard deviation of intercept	0.0323
Correlation coefficient (r^2^)	0.9991

**Tab. 4:** Precision and accuracy of the determination method of memantine hydrochloride in standard solutions (*n*=9; 3 sets for 3 days)

Concentration added (μg mL^−1^)	Concentration calculated (mean±S.D.) (μg mL^−1^)	R.S.D. (%)	Error (%)
Within-day (n=3)			
1.00	1.04±0.01	0.82	3.71
6.00	5.92±0.03	0.58	−1.40
12.00	11.99±0.06	0.46	−0.03
Between-day (n=9)			
1.00	1.05±0.01	0.05	5.13
6.00	5.85±0.06	0.95	−2.11
12.00	11.98±0.08	0.67	−0.10

**Tab. 5:** The Influence of small changes in pH and composition of mobile phase (method robustness)

Mobile phase composition	t_R_	Peak area
Acetonitrile–buffer pH=2.3 (73 : 27)	4.907	66.1
Acetonitrile–buffer pH=2.3 (75 : 25)	4.082	65.9
Acetonitrile–buffer pH=2.3 (77 : 23)	3.500	66.6
Acetonitrile–buffer pH=2.5 (73 : 27)	4.975	65.8
Acetonitrile–buffer pH=2.5 (75 : 25)	4.145	65.8
Acetonitrile–buffer pH=2.5 (77 : 23)	3.554	66.3
Acetonitrile–buffer pH=2.7 (73 : 27)	4.901	65.9
Acetonitrile–buffer pH=2.7 (75 : 25)	4.096	66.1
Acetonitrile–buffer pH=2.7 (77 : 23)	3.484	66.3

t_R_: retention time (min).
